# Parity-time phase transition in photonic crystals with $C_{6v}$ symmetry

**DOI:** 10.1038/s41598-020-72716-x

**Published:** 2020-09-25

**Authors:** Jeng-Rung Jiang, Wei-Ting Chen, Ruey-Lin Chern

**Affiliations:** Institute of Applied Mechanics, National Taiwan University, Taipei, 106 Taiwan

**Keywords:** Photonic crystals, Nanophotonics and plasmonics, Topological insulators

## Abstract

We investigate the parity-time (PT) phase transition in photonic crystals with $C_{6v}$ symmetry, with balanced gain and loss on dielectric rods in the triangular lattice. A two-level non-Hermitian model that incorporates the gain and loss in the tight-binding approximation was employed to describe the dispersion of the PT symmetric system. In the unbroken PT phase, the double Dirac cone feature associated with the $C_{6v}$ symmetry is preserved, with a frequency shift of second order due to the presence of gain and loss. The helical edge states with real eigenfrequencies can exist in the common band gap for two topologically distinct lattices. In the broken PT phase, the non-Hermitian perturbation deforms the dispersion by merging the frequency bands into complex conjugate pairs and forming the exceptional contours that feature the PT phase transition. In this situation, the band gap closes and the edge states are mixed with the bulk states.

## Introduction

Topological insulators (TIs) are a new phase of matter that are insulating in the bulk but feature conducting states on the surfaces^1,2^. The quantum Hall state^3^, a two-dimensional electron gas in a static magnetic field, is a well known topological phase with broken time-reversal (TR) symmetry. The quantum spin Hall (QSH) state^4–6^ belongs to a different topological class that preserves the TR symmetry, in which no magnetic field is required and the spin-orbit interaction is responsible for the topological character. The theoretical concepts developed in the QSH states were then generalized to three-dimensional TIs^7^ as a novel state of quantum matter.

Inspired by the discovery of TIs, there has been a surge of interest in the study of topological phases in photonic systems^8–16^. The most intriguing property of a topological phase is the emergence of a pair of helical edge states that are protected by TR symmetry^17^. The two states with opposite spin counterpropagate at a given edge without backscattering even in the presence of disorder. The existence of edge states is determined by the topological structure of the bulk states, characterized by the ${Z}_{2}$ topological invariant^18^ or spin Chern number^19^. The edge states come in Kramers doublet, which are doubly degenerate and TR partners to each other. In the presence of spin-orbit interaction, the degeneracy of the Kramers pair is lifted and the phase becomes topologically nontrivial. The Kramers degeneracy theorem, however, is usually valid for a TR invariant system with spin 1/2^17^ and cannot readily apply to the photonic system with spin 1, unless additional symmetry has been imposed in the system.

In the bianisotropic metacrystals–supperlattices of metamaterials^12^, the ’spin’-degenerate condition is introduced to form two *pseudospin* states by the linear combinations of transverse magnetic (TM) and transverse electric (TE) waves. The magnetoelectric coupling engineered in the metamolecules emulates the spin-orbit interaction in the photonic system, which is manifest on the entanglement between the phase relationship in waves (spin state) and the polarization of dipole moment (orbital state). In the dielectric photonic crystals with $C_{6v}$ symmetry, the combinations of doubly degenerate $E_1$ and $E_2$ modes^20^ form two pairs of pseudospin states, which are referred to as the *p* and *d* orbitals. The two modes can be accidentally degenerate at the Brillouin zone center, where the band dispersion is described by the double Dirac cone with four-fold degeneracy^21^. Once the band inversion occurs between the *p* and *d* orbitals, the photonic lattice exhibits a nontrivial topological phase^13^. The topological feature associated with the double Dirac cone has been analyzed in various photonic crystals with $C_{6v}$ symmetry, composed of cylinder arrays^13,22,23^, triangular holes^24,25^, and core shells^26^.

The photonic topological system can be described by the effective Hamiltonian consisting of two subsystems for the pseudospin states with opposite helicity^12,13,15,27,28^. In the presence of loss, the effective Hamiltonian of the system is non-Hermitian and the eigenvalue spectrum is no longer real^29–31^. The effect of loss is typically mitigated or compensated by gain. By judiciously incorporating gain and loss in the system, the non-Hermitian Hamiltonian has a real spectrum as long as it is parity-time (PT) symmetric, that is, the Hamiltonian commutes with the combined parity-inversion and TR operator^32^. The PT symmetry, however, is *broken* when the magnitude of balanced gain and loss is increased above a threshold, called the exceptional point^33^. In the broken PT phase, the eigenvalues of the system cease to be real and appear as complex conjugate pairs. At the point of degeneracy, the PT symmetry is spontaneously broken in the presence of an infinitesimal amount of gain and loss^34^. For the conical band structure of a Dirac cone resulting from accidental degeneracy^35^ or lattice symmetry^36,37^, the complex eigenvalues of the system are deformed into a two-dimensional flat band enclosed by an exceptional ring^35^ or exceptional contour^38,39^.

The existence of topological edge states in PT symmetric non-Hermitian systems has been a topic of ongoing discussion^29,40,41^, with regard to the complex eigenvalues of the states. The topological nature of the edge states was explored in a non-Hermitian system with the dimer model^42,43^, where the topologically distinct phases correspond to two inequivalent dimerization patterns. The topological edge states in PT symmetric systems was also demonstrated in one-dimensional waveguide arrays^44^, where the spectrum exhibits entirely real eigenvalues. More recently, the PT phase transition of the valley edge states was studied in two-dimensional honeycomb lattices^45^. The topological edge states are present even when their energies might be complex valued, as long as the bulk band gap is not closed.

In the present work, we investigate the PT phase transition in photonic crystals with $C_{6v}$ symmetry, with balanced gain and loss on dielectric rods in the triangular lattice. The photonic system is described by an effective Hamiltonian based on the tight binding model for the triangular lattice with $C_{6v}$ symmetry^21^. By treating the gain and loss as a non-Hermitian perturbation to the system^40,41^, the Hamiltonian can be separated into two parts^46^. The Hermitian part is in a similar form as the Bernevig–Hughes–Zhang (BHZ) model, which exhibits the dispersion of a double Dirac cone when the system is in the unbroken phase of PT symmetry. The skew-Hermitian part can deform the dispersion by merging the bands into complex conjugate pairs and forming the exceptional contours near the Dirac point. By exploiting the symmetry properties of the underlying triangular lattice, the exceptional point that features the PT symmetry breaking can be analyzed through the eigenfrequency of the non-Hermitian system.

In the unbroken PT phase, the edge states lie inside the common gap between the *p* and *d* bands for two topologically distinct lattices with PT symmetry, where the band inversion occurs in one of the lattices. The edge states can have a purely real eigenvalue spectrum and retain the helical nature as in the Hermitian case. The two states with opposite helicity counterpropagate at a given edge and are robust against scattering from disorder. In particular, the edge states become complex in frequency as the strength of balanced gain and loss increases, even when the bulk states are still real. In the broken PT phase, the bulk bands overlap at their quadratic mean frequency and the band gap closes. In this situation, the edge states are mixed with the bulk bands.

**Figure 1:**
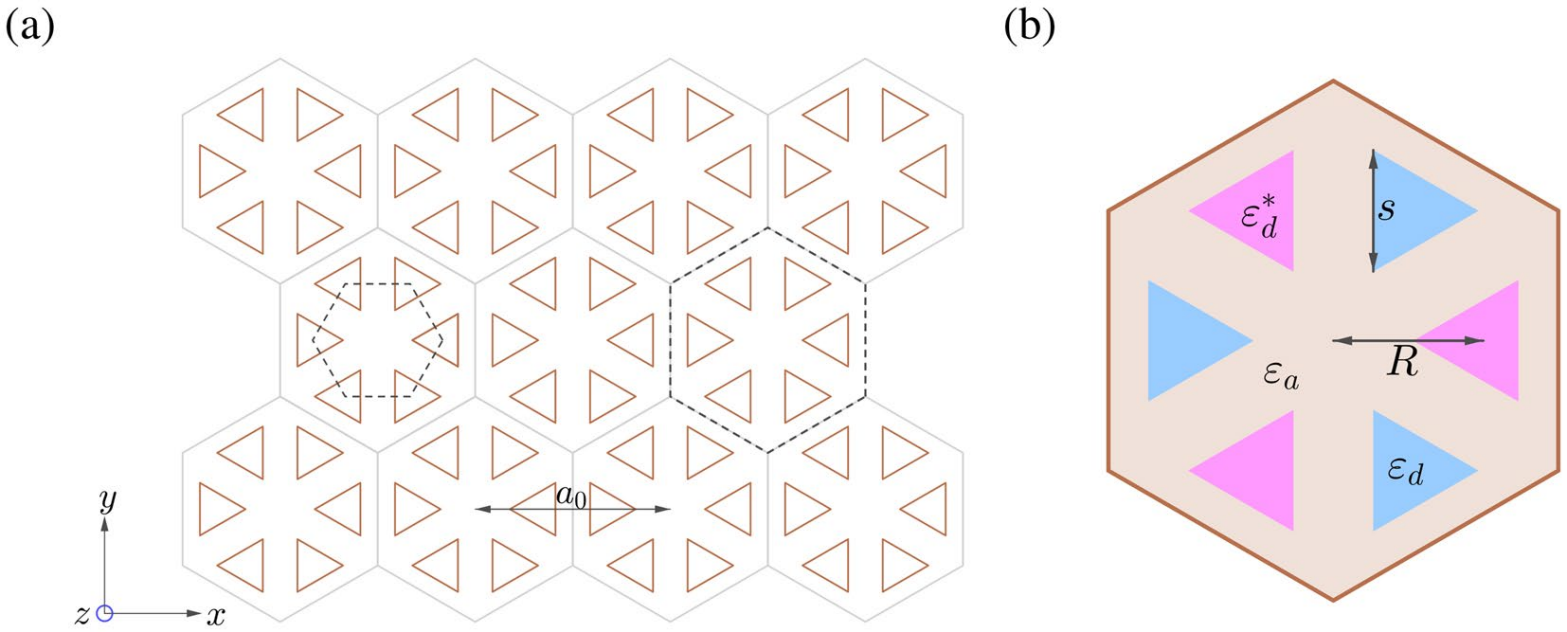
Schematic diagram of the PT symmetric photonic crystal with $C_{6v}$ symmetry. (**a**) triangular lattice, (**b**) unit cell, where $a_{0}$ is the lattice constant, *R* is the distance from the unit cell center to the centroid of each triangular rod of side length *s*, and $\varepsilon _{d}$ ($\varepsilon _{d}^*$) and $\varepsilon _a$ are the dielectric constants of triangular rods with gain (loss) and background medium, respectively.

## Results

### Two-level non-Hermitian model

Consider a two-dimensional photonic crystal consisting of equilateral triangular rods arranged as hexagonal clusters in the triangular lattice, as schematically shown in Fig. 1a. The wave equation in terms of the time-harmonic magnetic field $\mathbf {H}(\mathbf {r})$ (with the time convention $e^{-i\omega t}$) is given by

1 $$\begin{aligned} {{{\mathscr {L}}}}\mathbf {H}(\mathbf {r}) \equiv \nabla \times \left[ {\frac{1}{{\varepsilon (\mathbf {r})}}\nabla \times \mathbf {H}(\mathbf {r})} \right] = \frac{{\omega ^{2}}}{{{c^2}}}{{\mathbf {H}}}({\mathbf {r}}), \end{aligned}$$

where $\varepsilon (\mathbf {r})$ is the complex dielectric function. Assume that the lattice possesses the parity-time (PT) symmetry, that is, the Hamiltonian commutes with the combined PT operator: $({\mathscr {P}}{\mathscr {T}}){{\mathscr {L}}} ={{\mathscr {L}}}({\mathscr {P}}{\mathscr {T}})$, where ${\mathscr {P}}$ and ${\mathscr {T}}$ are the parity-inversion and TR operators, respectively. A necessary condition for the PT symmetry to hold is that the dielectric function satisfies $\varepsilon (\mathbf {r})=\varepsilon ^{*}(-\mathbf {r})$, where $\varepsilon (\mathbf {r})\equiv \varepsilon _{r}(\mathbf {r}) +i\varepsilon _{i}(\mathbf {r})$ with both $\varepsilon _{r}$ and $\varepsilon _{i}$ being real. For a triangular lattice composed of dielectric rods, the PT symmetry is established by an antisymmetric arrangement of gain and loss on the rods in the unit cell (cf. Fig 1b). Denote $\gamma$ and $g(\mathbf {r})$ the strength and distribution of gain and loss, respectively, that is, $\varepsilon _{i}(\mathbf {r}) \equiv \gamma g(\mathbf {r})$, where $\gamma \equiv \mathop {\max }\limits _{\mathbf {r}} \left| \varepsilon _{i} (\mathbf {r})\right|$ and $-1\le g(\mathbf {r})\le 1$. The gain and loss are applied such that $g(\mathbf {r})=-g(-\mathbf {r})$ and the convention $\gamma \ge 0$ is adopted. In the present problem, we propose that $\varepsilon _i(\mathbf {r})$ changes sign by a rotation of $60^\circ$ about the unit cell center. If $\gamma$ is zero, ${\mathscr {L}}$ is a Hermitian operator and $\omega$ is always real^47^.

Suppose that $\gamma$ is small compared to the maximum of $|\varepsilon _{r} (\mathbf {r})|$, and $i\gamma g(\mathbf {r})$ is treated as a small perturbation to the dielectric function^38^. Using the binomial expansion for $\frac{1}{\varepsilon (\mathbf {r})}$, the wave equation is approximated as

2 $$\begin{aligned} \nabla \times \left[ {\left( {\frac{1}{{{\varepsilon _r}({\mathbf{r}})}} -\frac{{i\gamma g({\mathbf{r}})}}{{\varepsilon _r^2({\mathbf{r}})}}} \right) \nabla \times {\mathbf{H}}({\mathbf{r}})} \right] = \frac{{{\omega ^2}}}{{{c^2}}}{\mathbf{H}}({\mathbf{r}}). \end{aligned}$$

In a two-level non-Hermitian model^46^, the operator ${\mathscr {L}}$ is separated into a Hermitian part ${{\mathscr {L}}}_{h} \equiv \nabla \times \frac{1}{\varepsilon _{r}(\mathbf {r})}\nabla \times$ and a skew-Hermitian part ${{\mathscr {L}}}_{s}\equiv -\nabla \times \frac{i\gamma g(\mathbf {r})}{\varepsilon ^{2}_{r}(\mathbf {r})} \nabla \times$. For a structure with $C_{6v}$ point group symmetry, there exist doubly degenerate $E_{1}$ and $E_{2}$ states, with the polynomial representations $\{x,y\}$ and $\{2xy,x^2-y^2\}$, respectively^48^. In a triangular lattice with $C_{6v}$ symmetry, the eigenmodes of the $E_{1}$ and $E_{2}$ symmetries are also identified at the Brilloin zone center (the $\Gamma$ point)^21^, which are referred to as the *p* and *d* orbitals, respectively^13^.

### Effective Hamiltonian

Based on the tight-binding approximation for the triangular lattice, the eigenfield around the *p* and *d* bands at the wave vector $\mathbf{k}$ near the $\Gamma$ point is expressed as^21^

3 $$\begin{aligned} {{\mathbf {H}}_{\mathbf {k}}}({\mathbf {r}}) = \sum \limits _{m = 0}^6 {{e^{i{\mathbf {k}} \cdot {{\mathbf {r}}_m}}}\sum \limits _{j = 1}^4 {{\alpha _j}{{\mathbf {H}}^{(j)}}({\mathbf {r}} - {{\mathbf {r}}_m})} }, \end{aligned}$$

where ${{\mathbf {H}}^{(1)}}$ and ${{\mathbf {H}}^{(2)}}$ (${{\mathbf {H}}^{(3)}}$ and ${{\mathbf {H}}^{(4)}}$) are the normalized magnetic fields of the $E_1$ ($E_2$) state for a *single* unit structure with $C_{6v}$ symmetry and $\alpha _j$ ($j=1,2,3,4$) is the weighting coefficient. Here, $m=0$ denotes the center cell, $m = 1,2, \ldots ,6$ its nearest neighboring cells with the centers at $\mathbf{r}_m$, and ${e^{i{\mathbf {k}} \cdot {{\mathbf {r}}_m}}}$ is included to satisfy the Bloch theorem: $\mathbf{H}_\mathbf{k}(\mathbf{r}+\mathbf{R})=e^{i\mathbf{k}\cdot \mathbf{R}}{} \mathbf{H}_\mathbf{k}(\mathbf{r})$ on the lattice. The states $\mathbf{H}^{(1)}$, $\mathbf{H}^{(2)}$, $\mathbf{H}^{(3)}$, and $\mathbf{H}^{(4)}$ form a basis for the four-band subsystem, which is denoted as $\left\{ p_{x}, p_{y}, d_{2xy}, d_{x^{2} - y^{2}}\right\}$. Using Eq. (3) in the eigensystem at the frequency $\omega _\mathbf {k}$ with the wave vector $\mathbf{k}$:

4 $$\begin{aligned} {\mathscr {L}}{\mathbf {H}_\mathbf {k}}(\mathbf {r}) = \frac{{\omega _\mathbf {k}^2}}{{{c^2}}}{\mathbf {H}_\mathbf {k}}(\mathbf {r}), \end{aligned}$$

and taking the inner product with ${{\mathbf {H}}^{(i)}}$ on both sides, the condition of a nontrivial solution of $\alpha _j$ [cf. Eq. (3)] gives the following secular equation (see Methods A):

5 $$\begin{aligned} \left| {{{\mathscr {H}}} - \frac{{\omega _\mathbf {k}^2}}{{{c^2}}}{{\mathscr {I}}}} \right| = 0, \end{aligned}$$

where ${{\mathscr {H}}}$ is the Hamiltonian of the four-band system and ${{\mathscr {I}}}$ is the $4\times 4$ identity matrix.

In the present two-level model, the effective Hamiltonian for the Hermitian operator ${\mathscr {L}}_{h}$ on the basis $\left\{ p_{x}, p_{y}, d_{2xy}, d_{x^{2} - y^{2}}\right\}$ in the linear order of $\mathbf{k}$ is given by (see Methods C.1)

6 $$\begin{aligned} {{\mathscr {H}}}_{h} = \left[ {\begin{array}{cccc} {\frac{{\omega _{p}^2}}{{{c^2}}}} &{} {0} &{} iAk_{y} &{} iAk_{x} \\ { 0} &{} {\frac{{\omega _{p}^2}}{{{c^2}}}} &{} iAk_{x} &{} { - iAk_{y}} \\ {- iAk_{y}} &{} {- iAk_{x}} &{} {\frac{{\omega _{d}^2}}{{{c^2}}}} &{} {0} \\ {- iAk_{x}} &{} {iAk_{y}} &{} { 0} &{} {\frac{{\omega _{d}^2}}{{{c^2}}}} \\ \end{array} } \right] , \end{aligned}$$

where *A* is a real quantity^13^. The Hermitian Hamiltonian ${{\mathscr {H}}}_h$ has the same form as in the photonic structure with $C_{6v}$ symmetry^26,49^. The effective Hamiltonian for the skew-Hermitian operator ${\mathscr {L}}_{s}$ on the same basis is given by (see Methods C.2)

7 $$\begin{aligned} {{\mathscr {H}}}_{s} = \left[ {\begin{array}{cccc} 0 &{} 0 &{} {i\gamma {N_1}} &{} {i\gamma {N_2}} \\ 0 &{} 0 &{} { - i\gamma {N_2}} &{} {i\gamma {N_1}} \\ {i\gamma {N_1}} &{} { -i\gamma {N_2}} &{} 0 &{} 0 \\ {i\gamma {N_2}} &{} {i\gamma {N_1}} &{} 0 &{} 0 \\ \end{array}} \right] , \end{aligned}$$

where $N_1$ and $N_2$ are real quantities. The effective Hamiltonian for the non-Hermitian operator ${\mathscr {L}}$, given by ${{\mathscr {H}}}={{\mathscr {H}}}_{h}+{{\mathscr {H}}}_{s}$, *has a block form of the PT symmetric matrix with a generic* (2, 2) *parity operator*^50^. A similar form of the non-Hermitian Hamiltonian was also present for the photonic structures with $C_{4v}$ symmetry^38^.

### PT phase transition

Based on Eqs. (5), (6), and (7), the eigenfrequency of the four-band non-Hermitian system can be solved to give the following formula:

8 $$\begin{aligned} \frac{{\omega _\mathbf{k}^2}}{{{c^2}}} = \frac{1}{2}\left( {\frac{{\omega _p^2}}{{{c^2}}} + \frac{{\omega _d^2}}{{{c^2}}}} \right) \pm \frac{1}{2}\sqrt{{{\left( {\frac{{\omega _p^2}}{{{c^2}}} - \frac{{\omega _d^2}}{{{c^2}}}} \right) }^2} +4{A^2}\left( {k_x^2 + k_y^2} \right) - 4\gamma ^{2} \left( {N _1^2 + N _2^2} \right) }, \end{aligned}$$

which are double roots for either $+$ or − sign. For $\gamma =0$, the doubly degenerate *p* and *d* bands have real eigenvalues as a consequence of the Hermitian property of ${{\mathscr {H}}}_h$. In particular, a four-fold degeneracy may occur when the *p* and *d* bands are *accidentally degenerate* ($\omega _p=\omega _d$) at the $\Gamma$ point, near which the dispersion is characterized by a double Dirac cone^21^. In general, $\omega _p\ne \omega _d$ and a gap between the *p* and *d* bands is opened. For $\gamma >0$, the eigenfrequencies of the four bands may not be real. Denote the discriminant of the square root in Eq. (8) as $\Delta \equiv {{\left( {{{\omega _p^2}}/{{{c^2}}} - {{\omega _d^2}}/{{{c^2}}}} \right) }^2} +4{A^2}\left( {k_x^2 + k_y^2} \right) - 4\gamma ^{2}\left( {N _1^2 + N _2^2}\right)$. If $\gamma$ is small enough such that $\Delta >0$, the eigenfrequencies are real and the PT symmetry is *unbroken*. On the other hand, if $\gamma$ exceeds a threshold value such that $\Delta <0$, the *p* and *d* bands are merged into complex conjugate pairs and the PT symmetry is *broken*. In this situation, the real parts of the *p* and *d* bands overlap at the their quadratic mean frequency: $\omega _0=\sqrt{\left( \omega _p^2+\omega _d^2\right) /2}$ and the gap in between is closed.

The exceptional point that features the transition between an unbroken and a broken PT phase is determined by the quadratic equation: $\Delta =0$. A collection of exceptional points that separate the regions of real eigenvalues and complex conjugate pairs form a contour in the wave vector space, the so-called exceptional contour^38^ or exceptional ring^35^. In the present problem, the PT phase transition is considered a balance between the strength of gain and loss ($\gamma$) and the deviation from the four-fold degeneracy either in frequency ($\omega _p^2-\omega _d^2$) or in wave number ($k_x^2+k_y^2$). In case the accidental degeneracy at the $\Gamma$ point is attained, where $\omega _p=\omega _d$ and $k_x=k_y=0$, we have $\Delta =- 4\gamma ^{2}\left( {N _1^2 + N _2^2}\right) <0$ for any nonzero $\gamma$ and the eigenfrequencies of the *p* and *d* bands are always complex conjugate pairs. In this situation, the PT symmetry is spontaneously broken in the presence of an infinitesimal amount of gain and loss^34^. The *thresholdless* PT symmetry breaking also occurs at the degenerate point with two-fold^36–38^ or three-fold^35^ degeneracy. It can be shown that the thresholdless PT symmetry breaking at the degenerate point is valid for a non-Hermitian system with finite decoupled bands (see Methods D).

If the non-Hermitian system is in the unbroken PT phase, that is, the eigenvalues are real, the perturbed frequency due to the presence of balanced gain and loss can be estimated by the perturbation method. For this purpose, a small number $\epsilon$ is defined as $\epsilon \equiv \gamma /\varepsilon _m$, where $\varepsilon _{m} \equiv \mathop {\max }\limits _{\mathbf {r}} \left| \varepsilon _{r} (\mathbf {r})\right|$. The frequency shift of the perturbed system is given by (see Methods E)

9 $$\begin{aligned} \Delta \omega = - \frac{c^2}{2\omega _0} \frac{\int _V \frac{i\gamma g(\mathbf{r})}{\varepsilon _{r}^2(\mathbf{r})} \left| \nabla \times \mathbf {H}_0 \right| ^{2} d {\mathbf {r}}}{\int _V \left| \mathbf {H}_0 \right| ^{2} d {\mathbf {r}}} + O\left( \epsilon ^2\right) , \end{aligned}$$

where $\mathbf {H}_0$ is the magnetic field at the eigenfrequency $\omega _0$ for the unperturbed system (without gain and loss). Note that the first term on the right-hand side of Eq. (9) is purely imaginary provided that $\omega _0$ is real (which is true when the unperturbed system is Hermitian). In the unbroken PT phase, the eigenvalues are real, which dictates that the first-order correction to the eigenfrequency is zero and *the frequency shift due to the presence of balanced gain and loss is of second order*: $\Delta \omega = O\left( \epsilon ^{2}\right)$. A similar form of the frequency shift can be obtained when the perturbation method is applied on the electric field: $\Delta \omega = - \frac{\omega _0}{2} \frac{\int _V {i\gamma g(\mathbf{r})} \left| \mathbf {E}_0 \right| ^{2} d {\mathbf {r}}}{\int _V \varepsilon _r(\mathbf{r}) \left| \mathbf {E}_0 \right| ^{2} d {\mathbf {r}}} + O\left( \epsilon ^2\right)$, where $\mathbf {E}_0$ is the electric field with the eigenfrequency $\omega _0$^51^.

**Figure 2:**
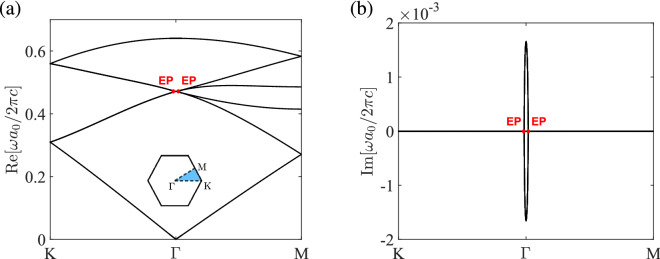
Thresholdless PT symmetry breaking at the four-fold degenerate point. (**a**,**b**) are the real and imaginary parts, respectively, of the band structure for the photonic crystal in Fig. 1 with $a_{0}/R = 3$, $s/a_0=0.317$, $\varepsilon _{a} = 1$, and $\varepsilon _{d} = 12 + 0.1i$. EP denotes the exceptional point.

## Discussion

### Broken PT phase

Figure 2 shows the band structure of TM modes along the high symmetry points of the first Brillouin zone for the PT symmetric photonic crystal with $a_0/R=3$. Here, the TM modes are characterized by the in-plane magnetic field components ($H_x$ and $H_y$) and the out-of-plane electric field component ($E_z$). If the gain and loss is not present ($\gamma =0$), the photonic crystal possesses $C_{6v}$ symmetry^21^. In this situation, the photonic crystal has two equivalent lattice descriptions (as a triangular lattice or a honeycomb lattice), which corresponds to the transition point between a trivial and a nontrivial topological phase^13^. For a small strength of gain and loss ($\varepsilon _d=12+0.1i$), the eigenfrequencies deviate from the lossless case up to $O\left( \epsilon ^2\right)$ [cf. Eq. (9)]. The real parts of the second to fifth bands (*p* and *d* bands) are nearly degenerate at the $\Gamma$ point (Fig. 2a), where the band dispersion is mainly described by a double Dirac cone [cf. Eq. (8)]. In the presence of balanced gain and loss, however small, the non-Hermitian system is spontaneously broken at the degenerate point^34^. The thresholdless PT symmetry breaking is manifest on the emergence of tiny imaginary parts of the *p* and *d* bands around the $\Gamma$ point (Fig. 2b).

**Figure 3:**
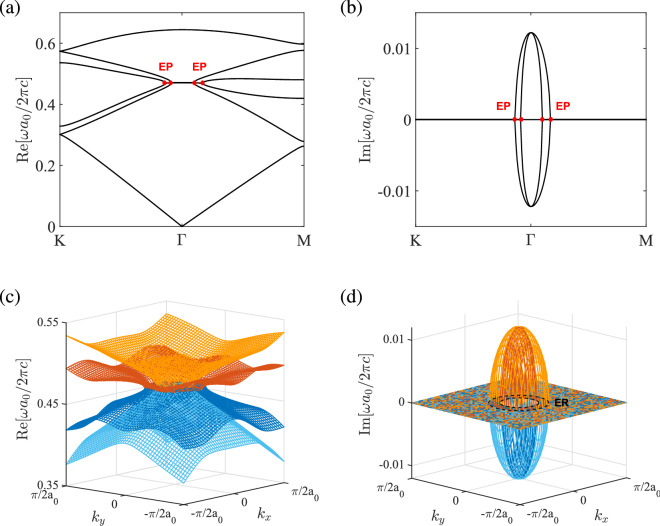
Bulk states in the broken PT phase. (**a**,**b**) are the real and imaginary parts, respectively, of the band structure for the photonic crystal in Fig. 1 with $a_{0}/R = 2.75$, $s/a_0=0.317$, $\varepsilon _{a} = 1$, and $\varepsilon _{d} = 12 + 1.8i$. (**c**,**d**) are the real and imaginary parts, respectively, of the dispersion surfaces for the *p* and *d* bands near the $\Gamma$ point. ER denotes the exceptional ring.

The band structure of the PT symmetric photonic crystal at $a_0/R=2.75$ for a larger strength of gain and loss ($\varepsilon _d=12+1.8i$) is shown in Fig. 3. The *p* and *d* bands merge into complex conjugate pairs in the region near the $\Gamma$ point, where the real parts of the eigenfrequencies overlap at their quadratic mean frequency (Fig. 3a). Meanwhile, the imaginary parts of the degenerate bands tend to split from the $\Gamma$ point to give two loops in the overlapping region (Fig. 3b). In this situation, the Dirac dispersion of the non-Hermitian system is deformed into flat bands near the center (Fig. 3c). The exceptional points that separate the regions of real eigenvalues (inside) and complex conjugate pairs (outside) form two contours in the wave vector space (Fig. 3d), which are characterized by the quadratic equation: $\Delta =0$ [cf. Eq. (8)]. Near the $\Gamma$ point, the dispersion surface is approximately isotropic with respect to $k_x$ and $k_y$, and the exceptional contours are roughly circles.

**Figure 4:**
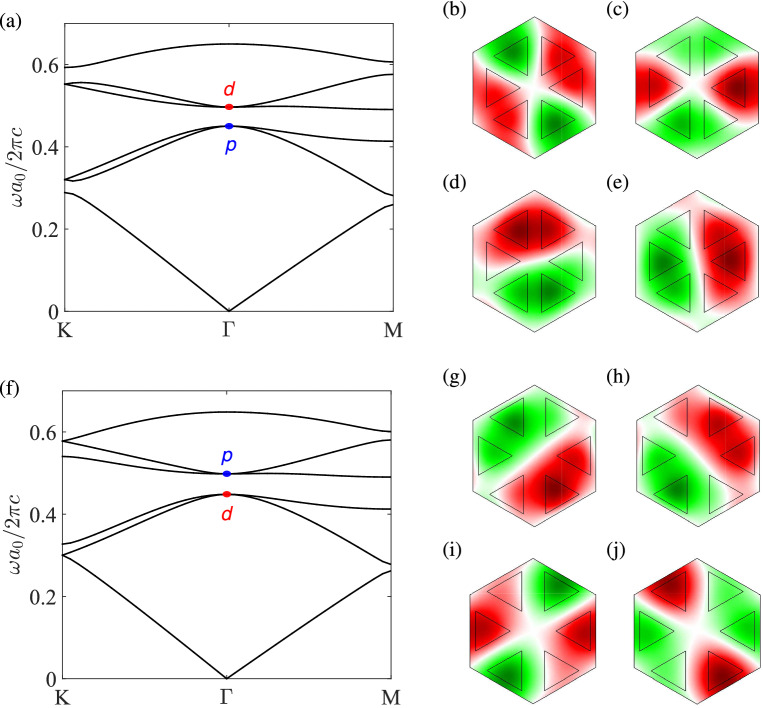
Bulk states in the unbroken PT phase. (**a**,**f**) are the band structures for the photonic crystals in Fig. 1 with $s/a_0=0.317$, $\varepsilon _{a} = 1$, and $\varepsilon _{d} = 12 + 0.6i$, corresponding to the trivial ($a_{0}/R = 3.35$) and nontrivial ($a_{0}/R = 2.75$) topological phases, respectively. (**b**–**e**) and (**g**–**j**) are the eigenfields ($\mathrm{Re}[E_z]$) at the $\Gamma$ point for the *p* bands (blue dot) and *d* bands (red dot) in (**a**,**f**), respectively.

### Unbroken PT phase

Figure 4 shows the band structures and eigenfields for the PT symmetric photonic crystal with $\varepsilon _d=12+0.6i$, where the bulk states are in the unbroken PT phase with real eigenvalues. For $a_0/R>3$, the *p* bands lie below the *d* bands ($\omega _p<\omega _d$) (Fig. 4a), that is, the dipole modes with odd parity (Fig. 4d,e) have the eigenfrequency lower than the quadrupole modes with even parity (Fig. 4b,c), and the photonic crystal has a trivial topological phase as in the Hermitian case. For $a_0/R<3$, on the other hand, the *p* bands lie above the *d* bands ($\omega _p>\omega _d$) (Fig. 4f), and the dipole modes (Fig. 4g,h) have the eigenfrequency higher than the quadrupole modes (Fig. 4i,j). In this situation, the band inversion (or parity inversion) between the *p* and *d* bands occurs, and the photonic crystal has a nontrivial topological phase as in the Hermitian case.

**Figure 5:**
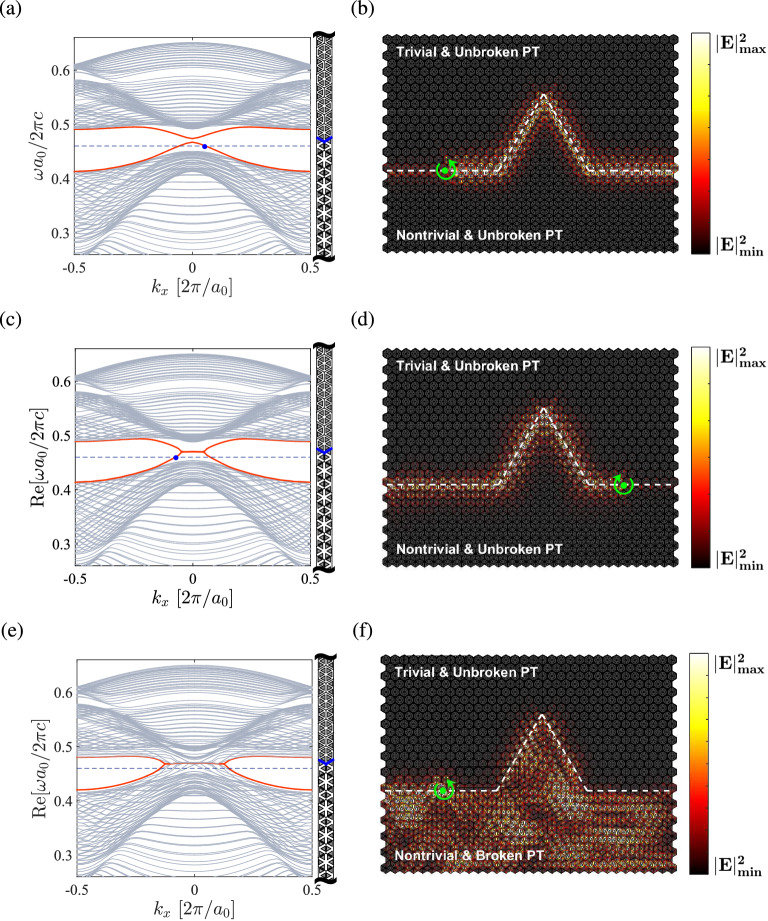
Edge states between two PT symmetric photonic crystals. (**a**,**c**,**e**) are the edge bands (in red color) at the interface between a trivial phase ($a_0/R=3.35$) with $\varepsilon _d=12+0.6i$ and a nontrivial phase ($a_0/R=2.75$) with $\varepsilon _d=12+0.6i$, $12+1.2i$, and $12+2i$, respectively. Light grays lines are the bulk bands for two concatenated crystals. Right vertical bar is the truncated supercell consisting of 20 cells on each side (interface in blue color). (**b**,**d**,**f**) are the surface wave propagations at the frequency marked by the blue dashed line in (**a**,**c**,**e**), respectively. White dashed line indicates the boundary between two crystals. Green dot denotes the circularly polarized magnetic dipole for exciting the surface wave.

### Edge states

Figure 5 shows the dispersions and wave propagations of the edge states at the interface between two PT symmetric photonic crystals, with a trivial topological phase ($a_0/R=3.35$) above the interface and a nontrivial phase ($a_0/R=2.75$) below. In Fig. 5a, both crystals are in the unbroken PT phase ($\varepsilon _d=12+0.6i$). For a small strength of gain and loss, the edge states, which lie inside the common band gap for the two crystals, can exhibit an entirely real spectrum along the $k_x$ direction. Notice that the edge states do not cross each other at the $\Gamma$ point and a small gap is opened in between, which means that the edge states may *not* be gapless^26^. This feature is attributed to the breaking of $C_{6v}$ symmetry at the boundary between two photonic crystals with different $a_0/R$, which is analogous to the effect of magnetic impurity that breaks the TR symmetry in a QSH system and opens a gap in the edge state^52^. As there is no point degeneracy in the edge states, the thresholdless PT symmetry breaking as in the bulk states does not occur. In Fig. 5b, a circularly polarized magnetic dipole composed of two perpendicular in-plane dipoles with $90^\circ$ phase difference is placed at the interface between a trivial phase (above the interface) and a nontrivial phase (below the interface) to excite the surface wave at a frequency inside the common gap (cf. blue dashed line in Fig. 5a), where the field is evanescent on either side of the interface. Excited by the dipole with ${H_0}e^{-i\omega t}\left( {\hat{x} + i\hat{y}} \right)$, the right-handed wave propagates unidirectionally toward the right, which is consistent with the surface band dispersion with a positive $k_x$ (cf. blue dot in Fig. 5a). In particular, the surface wave is able to bend around sharp corners without backscattering.

As the strength of balanced gain and loss increases, the gap in the edge states is reduced. At a certain value of $\gamma$, the edge states can be gapless in the real part, with the emergence of imaginary part at the crossing point. For a larger value of $\gamma$ in the nontrivial phase ($\varepsilon _d=12+1.2i$), the edge states merge into a complex conjugate pair, while the bulk band gap is still open, as shown in Fig. 5c. The edge states exhibit flat bands in the region near the $\Gamma$ point in a similar manner as the broken PT phase of the bulk states (cf. Fig. 3a). In Fig. 5(d), the left-handed wave is excited by ${H_0}e^{-i\omega t}\left( {\hat{x} - i\hat{y}} \right)$ at the same frequency (cf. blue dashed line in Fig. 5c) where the edge state has a real eigenfrequency (although the spectrum is not entirely real). The edge state propagates unidirectionally toward the left, which is consistent with the surface band dispersion with a negative $k_x$ (cf. blue dot in Fig. 5c). As in Fig. 5b, the surface wave is able to bend around sharp corners without backscattering. This feature holds if the larger value of $\gamma$ is given instead in the trivial phase. In Fig. 5a–d, the bulk states of both crystals are in the unbroken PT phase. The spin-polarized edge states retain the helical nature as in the Hermitian case, where the two states with opposite helicity (handedness) counterpropagate at a given edge and are robust against disorder.

For an even larger value of $\gamma$ in the nontrivial phase ($\varepsilon _d=12+2i$), where the PT symmetry is broken, the merged bulk bands occupy the original band gap region and the edge states are no longer separated from the bulk states, as shown in Fig. 5e. In this situation, the dipole source at the same frequency (cf. blue dashed line in Fig. 5e) excites not only the surface state (still having a real eigenvalue at the exciting frequency) but also the bulk states. The surface waves are mixed with the bulk waves that are scattered over the whole region on the lower side, as shown in Fig. 5f. If, on the other hand, the PT symmetry is broken in the trivial phase, the surface waves are to be mixed with the bulk waves on the upper side.

In conclusion, we have investigated the PT phase transition in photonic crystals with $C_{6v}$ symmetry, with balanced gain and loss on dielectric rods in the triangular lattice. The dispersion of the PT symmetric system was described by a two-level non-Hermitian Hamiltonian based on the tight-binding approximation, where the PT phase transition is featured with the exceptional contours in the wave vector space. In the unbroken PT phase, the double Dirac cone feature associated with the $C_{6v}$ symmetry is preserved, with a frequency shift of second order due to the presence of gain and loss. The helical nature of the edge states is manifest on the counterpropagation at the boundary and the robustness against disorder. In the broken PT phase, the bulk bands are merged into complex conjugate pairs and the edge states are no longer separated from the bulk states.

**Figure 6:**
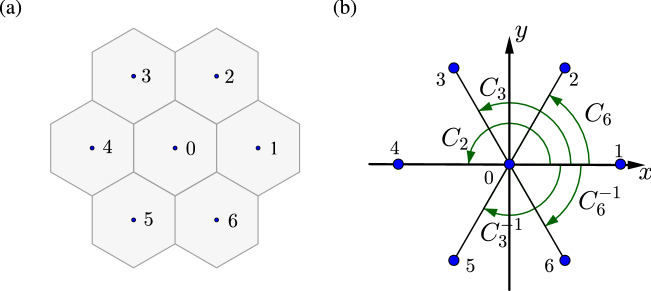
(**a**) Cell indices for the tight-binding approximation and (**b**) rotation operations in the $C_{6v}$ symmetry group.

## Methods

### A. Tight-binding model

Using the tight-binding approximation for the eigenfield [Eq. (3)]

10 $$\begin{aligned} {{\mathbf {H}}_{\mathbf {k}}}({\mathbf {r}}) = \sum \limits _{m = 0}^6 {{e^{i{\mathbf {k}} \cdot {{\mathbf {r}}_m}}}\sum \limits _{j = 1}^4 {{\alpha _j}{{\mathbf {H}}^{(j)}}({\mathbf {r}} - {{\mathbf {r}}_m})} }, \end{aligned}$$

in the eigensystem [Eq. (4)]

11 $$\begin{aligned} {\mathscr {L}}{\mathbf {H}_\mathbf {k}}(\mathbf {r}) = \frac{{\omega _\mathbf {k}^2}}{{{c^2}}}{\mathbf {H}_\mathbf {k}}(\mathbf {r}), \end{aligned}$$

and taking the inner product with ${{\mathbf {H}}^{(i)}({\mathbf {r}})}$ ($i=1,2,3,4$) on both sides, we have

12 $$\begin{aligned}&\sum \limits _{m = 0}^6 {e^{i{\mathbf {k}} \cdot {{\mathbf {r}}_m}}}\sum \limits _{j = 1}^4 {{\alpha _j}\int _V d {\mathbf {r}} \mathbf {H}^{(i)*}({\mathbf {r}}) \cdot {{\mathscr {L}}{\mathbf {H}}^{(j)}}({\mathbf {r}} - {{\mathbf {r}}_m})} \nonumber \\&\quad = \frac{{\omega _\mathbf {k}^2}}{{{c^2}}} \sum \limits _{m = 0}^6 {e^{i{\mathbf {k}} \cdot {{\mathbf {r}}_m}}}\sum \limits _{j = 1}^4 {{\alpha _j}\int _V d {\mathbf {r}} \mathbf {H}^{(i)*}({\mathbf {r}}) \cdot {{\mathbf {H}}^{(j)}}({\mathbf {r}} - {{\mathbf {r}}_m})}, \end{aligned}$$

where $\mathbf{r}_{0} = \left( 0,0 \right)$ and $\mathbf{r}_{m} = \left( {a_{0}\cos \frac{(m-1)\pi }{3}},{a_{0}\sin \frac{(m-1)\pi }{3}} \right)$ ($m = 1,2, \ldots ,6$) with $a_{0}$ being the lattice constant (cf. Fig. 6a). Let the $E_1$ and $E_2$ states be normalized as

13 $$\begin{aligned} \frac{1}{V}\int _V {{\mathbf {H}}^{(i)*}}({\mathbf {r}}) \cdot {{\mathbf {H}}^{(j)}}({\mathbf {r}})d{\mathbf {r}} = {\delta _{ij}}, \end{aligned}$$

where *V* is the area of the *single* unit structure. From the orthonormal condition on the unit cell as well as on the neighboring cells, Eq. (12) is recast into a system equation

14 $$\begin{aligned} \sum \limits _{j = 1}^{4} H_{ij}\alpha _{j} =\frac{{\omega _\mathbf {k}^2}}{{{c^2}}}\alpha _{i}, \end{aligned}$$

where

15 $$\begin{aligned} {H_{ij}} = \sum \limits _{m = 0}^6 {{e^{i{\mathbf {k}} \cdot {{\mathbf {r}}_m}}}I_m^{(ij)}}, \end{aligned}$$

with

16 $$\begin{aligned} I_m^{(ij)} = \frac{1}{V}\int _V {\mathbf {H}^{(i)*}}(\mathbf {r}) \cdot {{\mathscr {L}}}{\mathbf {H}^{(j)}}(\mathbf {r} - {\mathbf {r}_m})d \mathbf {r} \end{aligned}$$

being the *electromagnetic transfer integrals*^21^. A nontrivial solution of $\alpha _j$ requires that

17 $$\begin{aligned} \left| {{{\mathscr {H}}} - \frac{{\omega _\mathbf {k}^2}}{{{c^2}}}{{\mathscr {I}}}} \right| = 0, \end{aligned}$$

where ${{\mathscr {H}}}$ is a 4 $\times$ 4 matrix with the entries $H_{ij}$ ($i,j=1,2,3,4$) and ${{\mathscr {I}}}$ is the identity matrix.

### B. Matrix representations of the $E_1$ and $E_2$ modes in $C_{6v}$ symmetry group

For the structure with $C_{6v}$ point group symmetry, there exist doubly degenerate $E_1$ and $E_2$ states with the polynomial representations {*x*, *y*} and {2*xy*, $x^2 - y^2$}, respectively^48^. For the $E_1$ state, the matrix representations of the elements (used in the present study) in $C_{6v}$ symmetry group (cf. Fig. 6b) are given by^53^

18 $$\begin{aligned} E:\left[ {\begin{array}{cc} 1 &{} 0 \\ 0 &{} 1 \\ \end{array} } \right] \quad {C_6}:\left[ {\begin{array}{cc} {1/2} &{} {\sqrt{3} /2} \\ { - \sqrt{3} /2} &{} {1/2} \\ \end{array} } \right] \quad C_6^{ - 1}:\left[ {\begin{array}{cc} {1/2} &{} { - \sqrt{3} /2} \\ {\sqrt{3} /2} &{} {1/2} \\ \end{array} } \right] \nonumber \\ {C_2}:\left[ {\begin{array}{cc} { - 1} &{} 0 \\ 0 &{} { - 1} \\ \end{array} } \right] \quad {C_3}:\left[ {\begin{array}{cc} { - 1/2} &{} {\sqrt{3} /2} \\ { - \sqrt{3} /2} &{} { - 1/2} \\ \end{array} } \right] \quad C_3^{ - 1}:\left[ {\begin{array}{cc} { - 1/2} &{} { - \sqrt{3} /2} \\ {\sqrt{3} /2} &{} { - 1/2} \\ \end{array} } \right] . \end{aligned}$$

For the $E_2$ state, the corresponding matrix representations are given by

19 $$\begin{aligned} E:\left[ {\begin{array}{cc} 1 &{} 0 \\ 0 &{} 1 \\ \end{array} } \right] \quad {C_6}:\left[ {\begin{array}{cc} { - 1/2} &{} { - \sqrt{3} /2} \\ {\sqrt{3} /2} &{} { - 1/2} \\ \end{array} } \right] \quad C_6^{ - 1}:\left[ {\begin{array}{cc} { - 1/2} &{} {\sqrt{3} /2} \\ { - \sqrt{3} /2} &{} { - 1/2} \\ \end{array} } \right] \nonumber \\ {C_2}:\left[ {\begin{array}{cc} 1 &{} 0 \\ 0 &{} 1 \\ \end{array} } \right] \quad {C_3}:\left[ {\begin{array}{cc} { - 1/2} &{} {\sqrt{3} /2} \\ { - \sqrt{3} /2} &{} { - 1/2} \\ \end{array} } \right] \quad C_3^{ - 1}:\left[ {\begin{array}{cc} { - 1/2} &{} { - \sqrt{3} /2} \\ {\sqrt{3} /2} &{} { - 1/2} \\ \end{array} } \right] . \end{aligned}$$

### C. Effective Hamiltonian

Let the effective Hamiltonian ${\mathscr {H}}$ [cf. Eq. (17)] be divided into the Hermitian part ${{\mathscr {H}}}_h$ and the skew-Hermitian part ${{\mathscr {H}}}_s$ such that ${{\mathscr {H}}}={{\mathscr {H}}}_h+{{\mathscr {H}}}_s$.

### C.1 Hermitian part

The matrix entries for the Hermitian part ${{\mathscr {H}}}_h$ are given by [cf. Eqs. (15) and (16)]

20 $$\begin{aligned} {H^{(h)}_{ij}} = \sum \limits _{m = 0}^6 {{e^{i{\mathbf {k}} \cdot {{\mathbf {r}}_m}}}L_m^{(ij)}}, \end{aligned}$$

where

21 $$\begin{aligned} L_m^{(ij)} = \frac{1}{V}\int _V {\mathbf {H}^{(i)*}}(\mathbf {r}) \cdot {{\mathscr {L}}}_h{\mathbf {H}^{(j)}}(\mathbf {r} - {\mathbf {r}_m})d \mathbf {r}. \end{aligned}$$

In the vicinity of the $\Gamma$ point, the matrix entries can be approximated as

22 $$\begin{aligned} {H^{(h)}_{ij}} \approx \sum \limits _{m = 0}^6 {\left( 1 + i \mathbf{k} \cdot \mathbf{r}_m \right) L_m^{(ij)}}. \end{aligned}$$

By performing the operations in $C_{6v}$ symmetry group (cf. Methods B) on $L_m^{(ij)}$, ${H^{(h)}_{ij}}$ can be greatly simplified as listed in Ref.^21^, and the Hermitian part ${{\mathscr {H}}}_{h}$ is given as

23 $$\begin{aligned} {{\mathscr {H}}}_{h} = \left[ {\begin{array}{cccc} {\frac{{\omega _{p}^2}}{{{c^2}}}} &{} {0} &{} iAk_{y} &{} iAk_{x} \\ {0} &{} {\frac{{\omega _{p}^2}}{{{c^2}}}} &{} iAk_{x} &{} { - iAk_{y}} \\ {- iAk_{y}} &{} {- iAk_{x}} &{} {\frac{{\omega _{d}^2}}{{{c^2}}}} &{} {0} \\ {- iAk_{x}} &{} {iAk_{y}} &{} { 0} &{} {\frac{{\omega _{d}^2}}{{{c^2}}}} \\ \end{array} } \right] , \end{aligned}$$

where $A=2\sqrt{3}a_{0}L_{2}^{(13)}$^21^, which is a real quantity^13^.

### C.2 Skew-Hermitian part

The matrix entries for the skew-Hermitian part ${{\mathscr {H}}}_s$ are given by [cf. Eqs. (15) and (16)]

24 $$\begin{aligned} {H^{(s)}_{ij}} = \sum \limits _{m = 0}^6 {{e^{i{\mathbf {k}} \cdot {{\mathbf {r}}_m}}}G_m^{(ij)}}, \end{aligned}$$

where

25 $$\begin{aligned} G_m^{(ij)} = \frac{1}{V}\int _V {\mathbf {H}^{(i)*}}(\mathbf {r}) \cdot {{\mathscr {L}}}_s{\mathbf {H}^{(j)}}(\mathbf {r} - {\mathbf {r}_m})d \mathbf {r}. \end{aligned}$$

As the magnetic fields of the basis states $\mathbf{H}^{(i)}$ ($i=1,2,3,4$) are highly localized in the single unit structure^24,54^, the field couplings between the neighboring cells will be much weaker than those in the same cell. In the vicinity of the $\Gamma$ point, Eq. (24) can therefore be approximated as $H^{(s)}_{ij} \approx G_0^{(ij)}$. From Eq. (25), we have

26 $$\begin{aligned} G_0^{(ji)*} = - G_0^{(ij)}. \end{aligned}$$

For an operation ${{\mathscr {R}}}$ in the symmetric group (in terms of the matrix representation in Methods B), it can be shown that^20,21^

27 $$\begin{aligned} G_0^{(ij)} = \frac{1}{V}\int _V d \mathbf {r}{\left[ {{\mathscr {R}}} \mathbf {H}^{(i)*}\right] }(\mathbf {r}) \cdot {{\mathscr {R}}}{{\mathscr {L}}}_s {\left[ {{\mathscr {R}}}\mathbf {H}^{(j)}\right] }(\mathbf {r}), \end{aligned}$$

where $\left[ {{{\mathscr {R}}}{\mathbf {H}^{(i)}}} \right] (\mathbf {r}) \equiv {{\mathscr {R}}}{\mathbf {H}^{(i)}} \left( {{{{\mathscr {R}}}^{ - 1}}\mathbf {r}} \right)$. In the present PT symmetric configuration, the real part of the dielectric function satisfies $\left[ {{\mathscr {R}}}\varepsilon _{r} \right] (\mathbf {r}) = \varepsilon _{r} (\mathbf {r})$, while the imaginary part changes sign under the $C_{6}$ operation (rotation by $60^\circ$):

28 $$\begin{aligned} \left[ C_{6}\varepsilon _{i} \right] (\mathbf {r}) = - \varepsilon _{i} (\mathbf {r}). \end{aligned}$$

Using the $C_2$ operation for the $E_1$ state [cf. Eq. (18)] on Eq. (27), we have

29 $$\begin{aligned} G_0^{(ij)} = 0 \end{aligned}$$

for $i, j = 1, 2$. Likewise, using the $C_2$ operation for the $E_2$ state [cf. Eq. (19)] on Eq. (27), we have

30 $$\begin{aligned} G_0^{(ij)} = 0 \end{aligned}$$

for $$i, j = 3, 4$$.

Let ${\mathscr {P}}_{0}$ be the matrix representation of the parity operator ${\mathscr {P}}$ on the basis $\left\{ p_{x}, p_{y}, d_{2xy}, d_{x^{2} - y^{2}} \right\}$. Based on the $C_2$ operation for the $E_1$ and $E_2$ states [cf. Eqs. (18) and (19)], we have

31 $$\begin{aligned} {\mathscr {P}}_{0} = \left[ {\begin{array}{cccc} { - 1} &{} 0 &{} 0 &{} 0 \\ 0 &{} { - 1} &{} 0 &{} 0 \\ 0 &{} 0 &{} 1 &{} 0 \\ 0 &{} 0 &{} 0 &{} 1 \\ \end{array}} \right] , \end{aligned}$$

which is a generic (2, 2) parity operator^50^. Since ${{\mathscr {H}}}_s$ commutes with ${{\mathscr {P}}}_0{{\mathscr {T}}}$ (a consequence of the PT symmetry for both ${\mathscr {H}}$ and ${{\mathscr {H}}}_h$), it can be shown that^50^

32 $$\begin{aligned} {\mathscr {P}}_{0} {{\mathscr {H}}}_{s}={{\mathscr {H}}}_{s}^{*} {\mathscr {P}}_{0} \end{aligned}$$

and ${{\mathscr {H}}}_s$ has the block form

33 $$\begin{aligned} {{{\mathscr {H}}}_s} = \left[ {\begin{array}{cc} {{A_{2 \times 2}}} &{} {i{B_{2 \times 2}}} \\ {i{C_{2 \times 2}}} &{} {{D_{2 \times 2}}} \\ \end{array}} \right] , \end{aligned}$$

where $A_{2 \times 2}$, $B_{2 \times 2}$, $C_{2 \times 2}$, and $D_{2 \times 2}$ are *real* matrices. This means that $G^{(ij)}_{0}$ and $G^{(ji)}_{0}$ are purely imaginary for $i = 1, 2$ and $j = 3, 4$. Using the $C_6$ operation in Eqs. (18), (19), and (28) on Eq. (27), we have

34 $$\begin{aligned} {G^{(13)}_{0}} = \frac{1}{4}{G^{(13)}_{0}} + \frac{{\sqrt{3} }}{4}{G^{(14)}_{0}} + \frac{{\sqrt{3} }}{4}{G^{(23)}_{0}} + \frac{3}{4}{G^{(24)}_{0}}. \end{aligned}$$

Using the $C_6^{-1}$ operation in Eqs. (18), (19), and (28) on Eq. (27), we have

35 $$\begin{aligned} {G^{(13)}_{0}} = \frac{1}{4}{G^{(13)}_{0}} - \frac{{\sqrt{3} }}{4}{G^{(14)}_{0}} -\frac{{\sqrt{3} }}{4}{G^{(23)}_{0}} + \frac{3}{4}{G^{(24)}_{0}}. \end{aligned}$$

From Eqs. (34) and (35), we further have

36 $$\begin{aligned} G^{(13)}_{0} = G^{(24)}_{0} \end{aligned}$$

and

37 $$\begin{aligned} G^{(14)}_{0} = - G^{(23)}_{0}. \end{aligned}$$

Since ${{\mathscr {L}}}_{s}\equiv -\nabla \times \frac{i\gamma g(\mathbf {r})}{\varepsilon ^{2}_{r}(\mathbf {r})}\nabla \times$, we may factor out $\gamma$ from $G^{(ij)}_{0}$ [cf. Eq. (25)] such that $G^{(13)}_{0} \equiv i{\gamma N_1}$ and $G^{(14)}_{0} \equiv i{\gamma N_2}$. Based on Eqs. (26), (29), (30), (36), and (37), the skew-Hermitian Hamiltonian ${{\mathscr {H}}}_s$ is written as

38 $$\begin{aligned} {{\mathscr {H}}}_{s} = \left[ {\begin{array}{cccc} 0 &{} 0 &{} {i\gamma {N_1}} &{} {i\gamma {N_2}} \\ 0 &{} 0 &{} { - i\gamma {N_2}} &{} {i\gamma {N_1}} \\ {i\gamma {N_1}} &{} { - i\gamma {N_2}} &{} 0 &{} 0 \\ {i\gamma {N_2}} &{} {i\gamma {N_1}} &{} 0 &{} 0 \\ \end{array}} \right] , \end{aligned}$$

where $N_1$ and $N_2$ are real quantities.

### D. Thresholdless PT symmetry breaking

Suppose that *n* frequency bands for a PT symmetric non-Hermitian system can be decoupled from the rest of the system^38^ and their eigenstates form a basis in the subsystem.

#### Proposition

If the *n* bands in the decoupled subsystem are degenerate at a specific point, then the subsystem is Hermitian at that point.

#### Proof

Let $\phi _i$ ($i=1,\ldots ,n$) be the independent eigenstates of the Hamiltonian ${\mathscr {H}}$ for the subsystem with the same eigenvalue $\lambda _0$, that is, ${{\mathscr {H}}} \phi _i =\lambda _{0} \phi _i$. Since any state in the subsystem can be written as a linear combination of the basis: $\sum _i c_i\phi _i$, we have ${{\mathscr {H}}} = \lambda _{0} {{\mathscr {I}}}$, where ${{\mathscr {I}}}$ is the $n\times n$ identity matrix. From ${{\mathscr {H}}} ={\mathscr {P}}{\mathscr {T}}{\mathscr {P}} {\mathscr {T}}{\mathscr {H}}={\mathscr {P}}{\mathscr {T}}{\mathscr {H}} {\mathscr {P}}{\mathscr {T}} = {\mathscr {P}}{\mathscr {T}}{\mathscr {H}} {\mathscr {T}}{\mathscr {P}}={\mathscr {P}}{\mathscr {H}}^*{\mathscr {P}} ={{\mathscr {P}}}{\lambda _0^*}{\mathscr {I}}{\mathscr {P}} =\lambda _{0}^{*}{{\mathscr {I}}}$, we know that $\lambda _{0}=\lambda _0^*$ and thus $\lambda _0$ is real and ${{\mathscr {H}}}$ is Hermitian. $\square$

By contraposition, if ${{\mathscr {H}}}$ is non-Hermitian then the eigenvalues of the PT symmetric subsystem cannot be all equal. At the *n*-fold degenerate point where *all* eigenvalues in the subsystem have the same real part, *some* eigenvalues should have nonzero imaginary parts (otherwise, they will be all equal). This means that the PT symmetry is broken at the degenerate point (without threshold) for the non-Hermitian system stated above.

### E. Perturbation method

Let the presence of gain and loss in the non-Hermitian system be regarded as a perturbation to the Hermitian system^40,41^. By defining a small number $\epsilon \equiv \gamma /\varepsilon _m$, where $\varepsilon _{m} \equiv \mathop {\max }\limits _{\mathbf {r}} \left| \varepsilon _{r} (\mathbf {r})\right|$, the eigensystem is written as

39 $$\begin{aligned} {{\mathscr {L}}} \mathbf {H} =({{\mathscr {L}}}_0 +\epsilon {{\mathscr {L}}}_1) \mathbf {H} = \frac{\omega ^2}{c^2} \mathbf {H}, \end{aligned}$$

where ${{\mathscr {L}}}_{0}={{\mathscr {L}}}_{h} = \nabla \times \frac{1}{\varepsilon _{r}(\mathbf {r})}\nabla \times$ and ${{\mathscr {L}}}_{1}=\frac{1}{\epsilon }{{\mathscr {L}}}_{s} =-\nabla \times \frac{i\varepsilon _m g(\mathbf {r})}{\varepsilon ^{2}_{r}(\mathbf {r})}\nabla \times$. Let $\mathbf {H}_0$ be the eigenfield at the frequency $\omega _0$ for the unperturbed system, that is, ${{\mathscr {L}}}_{0} \mathbf {H}_0 =\frac{\omega _0^2}{c^2} \mathbf {H}_0$. The eigenfield for the perturbed system is expanded in a series as $\mathbf{H}=\mathbf {H}_0 +\epsilon \mathbf {H}_{1} + \epsilon ^2\mathbf {H}_{2} + \cdots$. Likewise, the eigenfrequency is expanded as $\omega =\omega _0+\epsilon \omega _1+\epsilon ^2\omega _2+\cdots$. Using these expansions in Eq. (39), we have

40 $$\begin{aligned}&\left( {{\mathscr {L}}}_{0} + \epsilon {{\mathscr {L}}}_{1} \right) \left( \mathbf {H}_0 + \epsilon \mathbf {H}_{1} +\epsilon ^2\mathbf {H}_{2} + \cdots \right) \nonumber \\&\quad = \left( \frac{\omega _0^2}{c^2} + \epsilon \frac{\omega _{1}^{2}}{c^2} + \epsilon ^2\frac{\omega _{2}^{2}}{c^2} + \cdots \right) \left( \mathbf {H}_0 + \epsilon \mathbf {H}_{1} + \epsilon ^2\mathbf {H}_{2} + \cdots \right) . \end{aligned}$$

The perturbed eigenfield $\mathbf{H}$ is normalized according to $\left\langle {\mathbf {H}_0} | {\mathbf {H}} \right\rangle =\left\langle {\mathbf {H}_0} | {\mathbf {H}}_0 \right\rangle$, so that the orthogonality condition $\left\langle {\mathbf {H}_0} | {\mathbf {H}}_n \right\rangle =0$ holds for $n>0$. The first-order term of the eigenfrequency is given by

41 $$\begin{aligned} \frac{\omega _{1}^{2}}{c^{2}} = \frac{\left\langle {\mathbf {H}_0} |{{{\mathscr {L}}}_{1} \mathbf {H}_0} \right\rangle }{\left\langle {\mathbf {H}_0}|{\mathbf {H}_0} \right\rangle } = -\frac{\int _V \mathbf {H}_0^{*}\cdot \nabla \times \frac{i\varepsilon _{m}g(\mathbf{r})}{\varepsilon _{r}^2(\mathbf{r})} \nabla \times \mathbf {H}_0 d {\mathbf {r}}}{\int _V \mathbf {H}_0^{*}\cdot \mathbf {H}_0 d {\mathbf {r}}} = -\frac{\int _V \frac{i\varepsilon _{m}g(\mathbf{r})}{\varepsilon _{r}^2(\mathbf{r})} \left| \nabla \times \mathbf {H}_0\right| ^{2} d {\mathbf {r}}}{\int _V \left| \mathbf {H}_0\right| ^{2} d {\mathbf {r}}}. \end{aligned}$$

The first-order correction to the eigenfrequency is then obtained as

42 $$\begin{aligned} \Delta \omega = \sqrt{\omega _0^{2} + \epsilon \omega _{1}^{2}} -\omega _0 \approx \omega _0\left( 1 +\frac{\epsilon }{2} \frac{\omega _{1}^{2}}{\omega _0^{2}} \right) -\omega _0 = - \frac{c^2}{2\omega _0} \frac{\int _V \frac{i\gamma g(\mathbf{r})}{\varepsilon _{r}^2(\mathbf{r})} \left| \nabla \times \mathbf {H}_0 \right| ^{2} d {\mathbf {r}}}{\int _V \left| \mathbf {H}_0 \right| ^{2} d {\mathbf {r}}}. \end{aligned}$$
